# Identification of mutations, gene expression changes and fusion transcripts by whole transcriptome RNAseq in docetaxel resistant prostate cancer cells

**DOI:** 10.1186/s40064-016-3543-0

**Published:** 2016-10-24

**Authors:** Yuanjun Ma, Yali Miao, Zhuochun Peng, Johanna Sandgren, Teresita Díaz De Ståhl, Mikael Huss, Lena Lennartsson, Yanling Liu, Monica Nistér, Sten Nilsson, Chunde Li

**Affiliations:** 1 Department of Oncology-Pathology, Karolinska Institutet, Stockholm, Sweden; 2 Department of Obstetrics and Gynecology, Beijing University People’s Hospital, Beijing, China; 3 SciLifeLab (Science for Life Laboratory), Stockholm, Sweden; 4 Clinical Pathology/Cytology, Karolinska University Hospital, Stockholm, Sweden; 5 Department of Clinical Oncology, Karolinska University Hospital, Stockholm, Sweden

**Keywords:** Docetaxel resistance, Prostate cancer, RNAseq, Gene fusion, Mutation, Altered expression

## Abstract

Docetaxel has been the standard first-line therapy in metastatic castration resistant prostate cancer. The survival benefit is, however, limited by either primary or acquired resistance. In this study, Du145 prostate cancer cells were converted to docetaxel-resistant cells Du145-R and Du145-RB by in vitro culturing. Next generation RNAseq was employed to analyze these cell lines. Forty-two genes were identified to have acquired mutations after the resistance development, of which thirty-four were found to have mutations in published sequencing studies using prostate cancer samples from patients. Fourteen novel and 2 previously known fusion genes were inferred from the RNA-seq data, and 13 of these were validated by RT-PCR and/or re-sequencing. Four in-frame fusion transcripts could be transcribed into fusion proteins in stably transfected HEK293 cells, including MYH9-EIF3D and LDLR-RPL31P11, which were specific identified or up-regulated in the docetaxel resistant DU145 cells. A panel of 615 gene transcripts was identified to have significantly changed expression profile in the docetaxel resistant cells. These transcriptional changes have potential for further study as predictive biomarkers and as targets of docetaxel treatment.

**Electronic supplementary material:**

The online version of this article (doi:10.1186/s40064-016-3543-0) contains supplementary material, which is available to authorized users.

## Background

Most metastatic prostate cancers respond to androgen deprivation therapy (ADT) but eventually develop castration resistance and become metastatic castration resistant prostate cancers (mCRPC) about 24–36 months after the treatment start (Harris et al. 2009; Attar et al. 2009; Watson et al. 2010). mCRPC is the major cause of cancer death in prostate cancer patients. Median survival time of patients with mCRPCs is 16–18 months from the start of progression (Amaral et al. 2012). Docetaxel chemotherapy can further prolong the median overall survival by 3–5 months (Galsky et al. 2012). However, docetaxel resistance is a critical problem because half of patients will not respond to docetaxel treatment (intrinsic resistance), while the other half, which responds initially, become resistant ultimately (acquired resistance) (Tannock et al. 2004). Failure of docetaxel treatment has been thought to be caused by either intrinsic or acquired resistance.

Docetaxel is a member of taxane family and widely been used to treat mCRPC patients. Docetaxel induces cancer cell death by binding β-tubulin, stabilizing microtubule assembly, suppressing dynamics of individual micro-tubules in G2-M phase tumor cells and preventing disassembly (Yvon et al. 1999; Eisenhauer and Vermorken 1998). Despite a decade of clinical use, the mechanism of resistance to docetaxel has not been fully investigated and there are no clinically reliable biomarkers to predict the drug resistance. Limited data suggests that the resistance may be caused by the following mechanisms: (1) decreased drug concentration due to high expression of drug export pump proteins ABCB1, ABCB4, ABCC1 (Gottesman et al. 2002); (2) mutations in the drug targets (Berrieman et al. 2004); (3) inhibition of apoptotic pathways (Bhalla 2003); (4) altered expression profile of tubulins or microtubule-associated proteins (MAPs) (Seve and Dumontet 2008; Verrills et al. 2006). So far, only a few drugs have been developed with modest survival benefit in docetaxel resistant mCRPC.

This study applied next generation RNA sequencing (RNAseq) technology in combination with specific software (Ozsolak and Milos 2011) to determine gene expression changes, mutations and fusions in docetaxel sensitive cell lines versus docetaxel resistant cell lines. The comparison between these cell lines identified a panel of genes potentially involved in the development of docetaxel resistance. The clinical importance was further addressed by comparing with published RNA sequencing results in prostate cancer samples from patients.

## Results

### Mutations acquired in docetaxel resistant cell lines

**Fig. 1:**
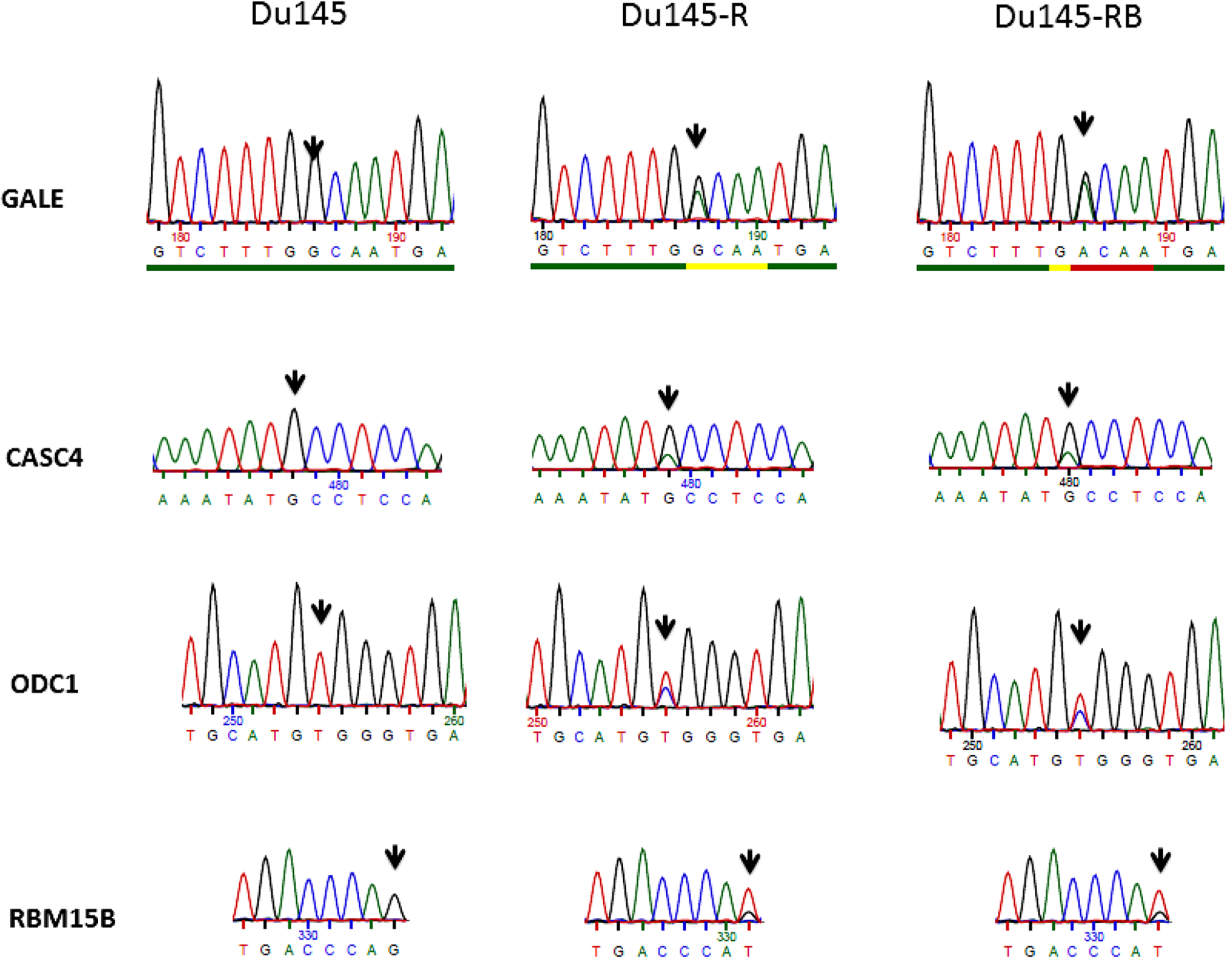
Mutation list validated by PCR followed by Sanger sequencing. *Black arrow* shows the mutation positions

We generated docetaxel resistant variants of Du145 prostate cancer cells as described in M&M. We used triplicates of each cell line (Du145, Du145-R and Du145-RB) for whole transcriptome RNA-sequencing and found 4864 mutations totally (Additional file 1). We compared TaxR (docetaxel resistant) and TaxS (docetaxel sensitive) cell lines to find mutations acquired after docetaxel treatment. Only mutations, which were absent in TaxS (Du145) but present in all Du145-R triplicates and Du145-RB triplicates, were chosen as “stably acquired mutations”. Forty-two such mutations were identified (Table 1) and 4 randomly selected mutations were validated by PCR followed by SANGER sequencing (Fig. 1).

**Table 1:** Mutations acquired after docetaxel treatment

Gene	Full name	Mutation		AA	Type	Non-synonymous	In prostate tumor samples?
GALE	UDP-galactose-4-epimerase	TTT G[G/A]C AAT	Single AA change	G > D	Damaging	Yes	yes
KIAA1522	KIAA1522	Deletion	Frameshift		NA	Yes	yes
ATP5F1	ATP synthase, H + transporting, mitochondrial Fo complex subunit B1	GCC AA[G/T] TGC	Single AA change	K > N	Damaging	Yes	
ZNF669	Zinc finger protein 669	GAA [C/T]AG TGT	Nonsense	Q > *	NA	Yes	yes
MDK	Midkine (neurite growth-promoting factor 2)	Deletion	Frameshift		NA	–	
DDX23	DEAD-box helicase 23	GCT G[A/T]C AAA	Single AA change	D > V	Damaging	Yes	yes
SFSWAP	Splicing factor, suppressor of white-apricot homolog	GAG [A/G]GG AGT	Single AA change	R > G	Damaging	Yes	yes
TJP1	Tight junction protein 1	CCA C[G/A]T TTT	Single AA change	R > H	Damaging	Yes	yes
CASC4	Cancer susceptibility candidate 4	AAT AT[G/A] CCT	Single AA change	M > I	Damaging	Yes	
MRPL28	Mitochondrial ribosomal protein L28	CAG G[A/G]C CCC	Single AA change	D > G	Damaging	Yes	yes
STUB1	STIP1 homology and U-box containing protein 1	ATC G[C/T]G AAG	Single AA change	A > V	Damaging	Yes	yes
UQCRC2	Ubiquinol-cytochrome C reductase core protein II	ACA A[A/C]A GGA	Single AA change	K > T	Damaging	Yes	yes
CHTF8	Chromosome transmission fidelity factor 8	CCC A[G/T]G TCA	Single AA change	R > M	Damaging	–	yes
KLHDC4	Kelch domain containing 4	GAC G[T/C]G TAT	Single AA change	V > A	Damaging	Yes	yes
SPATA20	Spermatogenesis associated 20	GTC [C/T]CT CAC	Single AA change	P > S	Damaging	Yes	yes
SMAD4	SMAD Family Member 4	Deletion	Frameshift		NA	Yes	yes
LSM14A	LSM14A MRNA processing body assembly factor	CAG T[C/T]C ATG	Single AA change	S > F	Damaging	Yes	yes
CALM3	Calmodulin 3 (phosphorylase kinase, delta)	GGG [G/A]AG AAG	Single AA change	E > K	NA	Yes	
MYADM	Myeloid-associated differentiation marker	TCC C[C/T]T CGG	Single AA change	P > L	Damaging	Yes	yes
ODC1	Ornithine decarboxylase 1	CAT G[T/C]G GGT	Single AA change	V > A	Damaging	Yes	yes
FOSL2	FOS like antigen 2	GAC [C/A]TG CAG	Single AA change	L > M	Damaging	Yes	yes
CYBRD1	Cytochrome B reductase 1	TTC [G/A]GG GCC	Single AA change	G > R	Damaging	Yes	
BOK	BCL2-related ovarian killer	GAC [T/C]GT GTG	Single AA change	C > R	Damaging	Yes	
ITCH	Itchy E3 ubiquitin protein ligase	AAT G[G/A]T GAA	Single AA change	G > D	Damaging	Yes	yes
DIP2A	Disco interacting protein 2 homolog A	AAC [G/A]TC TTC	Single AA change	V > I	Damaging	Yes	yes
BID	BH3 interacting domain death agonist	ACC [G/A]TA GCA	Single AA change	V > I	Damaging	Yes	yes
NUP210	Nucleoporin 210 kDa	ATA [G/T]CC TAC	Single AA change	A > S	Damaging	Yes	yes
HYAL2	Hyaluronoglucosaminidase 2	CTG [C/T]GA CCT	Nonsense	R > *	NA	Yes	
RBM15B	RNA binding motif protein 15B	ACC CA[G/T] CTG	Single AA change	Q > H	Damaging	Yes	yes
CTBP1	C-terminal binding protein 1	TCC A[C/T]G CAG	Single AA change	T > M	Damaging	Yes	yes
TACC3	Transforming acidic coiled-coil containing protein 3	AGC T[C/T]T TCC	Single AA change	S > F	Damaging	Yes	yes
AFAP1	Actin filament associated protein 1	TCA [G/C]AG GCC	Single AA change	E > Q	Damaging	Yes	yes
MCTP1	Multiple C2 and transmembrane domain containing 1	ATG G[G/T]C TCA	Single AA change	G > V	Damaging	Yes	yes
MAN2A1	Mannosidase alpha class 2A member 1	CTT A[T/A]C CAG	single aa change	I > N	Damaging	Yes	yes
C5orf15	Chromosome 5 open reading frame 15	Deletion	Frameshift		NA	Yes	
UIMC1	Ubiquitin interaction motif containing 1	GAA G[C/A]T AGG	Single AA change	A > D	Damaging	Yes	yes
MAML1	Mastermind like transcriptional coactivator 1	Deletion	Frameshift		NA	Yes	yes
C6orf141	Chromosome 6 open reading frame 141	CGG [G/T]GG CCT	Single AA change	G > W	Damaging	Yes	yes
CROT	Carnitine *O*-octanoyltransferase	TTT [T/C]CC AAA	Single AA change	S > P	Damaging	Yes	yes
CAPZA2	Capping actin protein of muscle Z-line alpha subunit 2	AGG A[A/C]G GAG	Single AA change	K > T	Damaging	Yes	yes
ASB6	Ankyrin Repeat and SOCS box containing 6	AAC [C/T]GC TTC	Single AA change	R > C	Damaging	Yes	yes
ABCA2	ATP binding cassette subfamily a member 2	GGC C[G/A]C TTC	Single AA change	R > H	Damaging	Yes	yes

Mutation and amino acid changing were showed in column 3 and 4. Mutation Type was analyzed by program SIFT and gained by setting cutoff as 0.05. ‘Damaging’ means that the substitution is predicted to affect protein function. *NA* not analyzed. The last column shows the connection between cell lines and tumor samples. It is labeled “yes” if mutation can be found in both cell line sequence and tumor sample sequence

By matching with previously published whole transcriptome analyses, we could identify that 34 of these genes had mutations in prostate cancer samples from patients (Table 1) (Robinson et al. 2015). For many genes, e.g. ABCB2, there are published data to support their importance in the development of drug resistance (Aberuyi et al. 2014; Rahgozar et al. 2014).

### Fusion transcript detection and validation

**Fig. 2:**
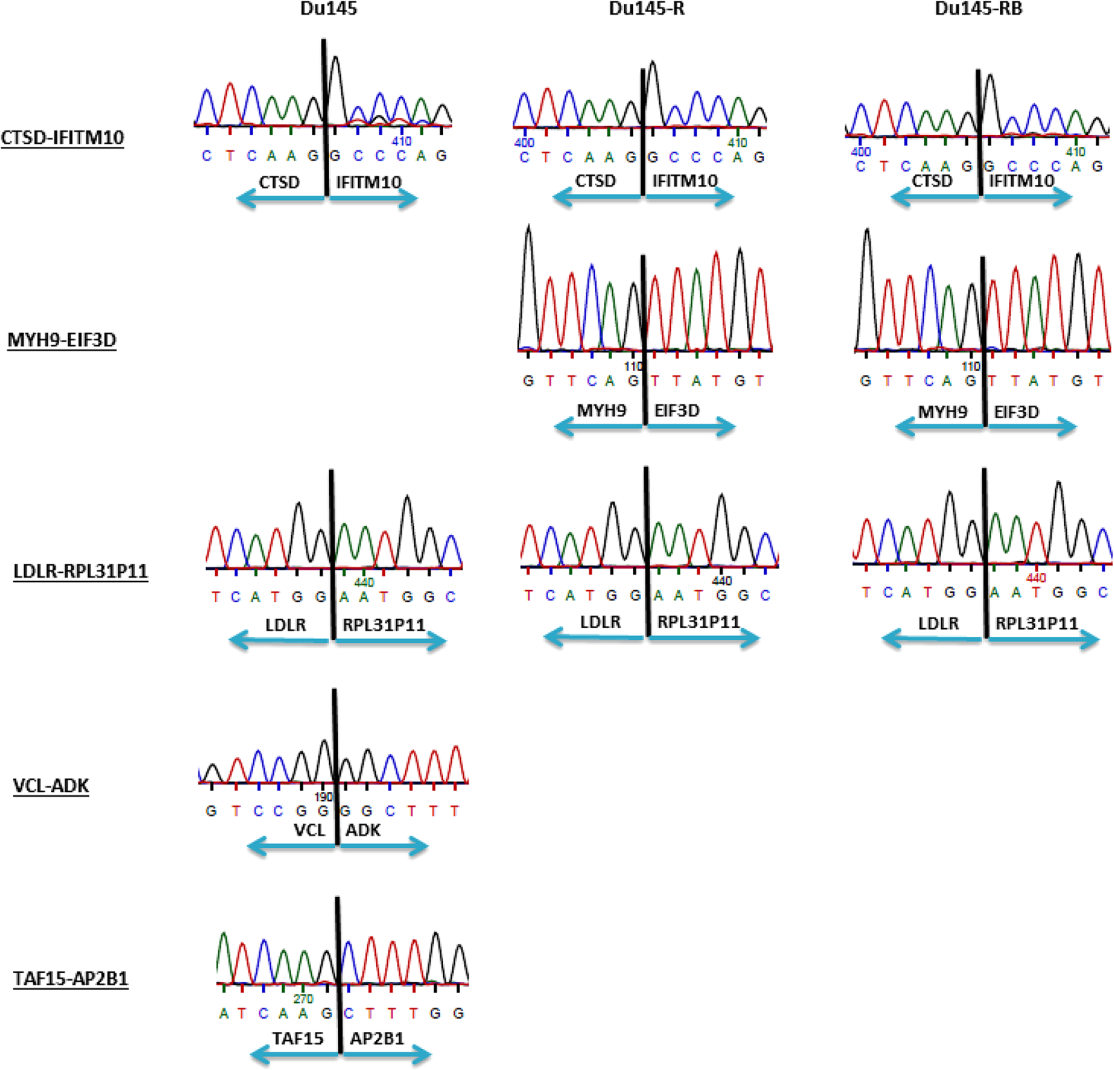
Sanger-sequencing validation of 5 fusion candidates discovered by NGS. *Black lines* indicates the fusion points between 2 genes

ChimeraScan software was employed to find fusions from RNAseq data. Selecting gene–gene pairs supported by two or more unique alignment reads provided an initial list of 48, 75 and 66 fusion candidates in DU145, Du145-R and Du145-RB cell lines respectively (Additional file 2). We validated all fusion candidates that had a ChimeraScan score above 5 in at least 1 out of 3 cell lines. Of 16 fusion candidates selected (Table 2), 13 (81.25 %) were verified by Reverse Transcription PCR (RT-PCR) with primers covering the fusion break points (Additional file 3), and 5 of validated genes were further verified by Sanger sequencing in the Du145, Du145-R and Du145-RB cell lines (Fig. 2). Two gene fusions had been found by previous studies: UBE2L3-KRAS (Wang et al. 2011) expressed in all three cell lines and TAF15-AP2B1 (http://54.84.12.177/PanCanFusV2/Fusions!fusion) specific expressed in Du145 (Additional file 3). The other fourteen fusions were novel discovered.

**Table 2:** Fusion transcripts identified by NGS and validated by PCR

5′ gene (full name)	5′ chr	3′ gene (full name)	3′ chr	Type	Verified	Express in TaxS	Express in TaxR
TAF15 (TATA-box binding protein associated factor 15)	17	AP2B1 (adaptor related protein complex 2 beta 1 subunit)	17	Read through	Yes	Yes	No
VCL (vinculin)	10	ADK (adenosine kinase)	10	Read through	Yes	Yes	No
MYH9 (myosin, heavy chain 9, non-muscle)	22	EIF3D (eukaryotic translation initiation factor 3 subunit D)	22	Read through	Yes	No	Yes
C14orf166 (chromosome 14 open reading frame 166)	14	SLC25A21 (solute carrier family 25 member 21)	14	Intra chromosomal	Yes	Yes	Yes
UBE2L3 (ubiquitin conjugating enzyme E2 L3)	22	KRAS (kirsten rat sarcoma viral oncogene homolog)	12	Inter chromosomal	Yes	Yes	Yes
LDLR (low density lipoprotein receptor)	19	RPL31P11 (ribosomal protein l31 pseudogene 11)	15	Read through	Yes	Yes	Yes (up regulated)
IGSF9B (immunoglobulin superfamily member 9B)	11	FAM177A1 (family with sequence similarity 177 member A1)	14	Inter chromosomal	No		
CTSD (cathepsin D)	11	IFITM10 (interferon induced transmembrane protein 10)	11	Read through	Yes	Yes	Yes (up regulated)
FLJ39739	1	BC065231	1	Intra chromosomal	Yes	Yes	Yes
LOC100286793	1	BC065231	1	Intra chromosomal	Yes	Yes	Yes
UBE2H (ubiquitin conjugating enzyme E2 H)	7	WIZ (widely interspaced zinc finger motifs)	19	Inter chromosomal	No		
SFPQ (splicing factor proline/glutamine-rich)	1	AL831889 (LOC100996496)	1	Read through	Yes	Yes	Yes
CADM4 (cell adhesion molecule 4)	19	ZNF428 (zinc finger protein 428)	19	Read through	Yes	Yes	Yes
GOLT1A (golgi transport 1A)	1	KISS1 (KiSS-1 metastasis-suppressor)	1	Read through	Yes	Yes	Yes
SRGAP2P2 (SLIT-ROBO Rho GTPase activating protein 2B)	1	SRGAP2 (SLIT-ROBO Rho GTPase activating protein 2)	1	Intra chromosomal	Yes	Yes	Yes (up regulated)
BTNL8 (butyrophilin like 8)	5	HMGA1 (high mobility group AT-Hook 1)	6	Inter chromosomal	No		

5′ end genes and their information were listed in column 1 and 2, and 3′ end genes in column 3 and 4. Read Through, new fusion gene can be read through when translated. Intrachromosomal, fusion partners come from same chromosomes. Interchromosomal, fusion formed between different chromosomes. Last column was marked ‘Yes’ if fusions can be verified in Du145, Du145-R or Du145-RB

**Fig. 3:**
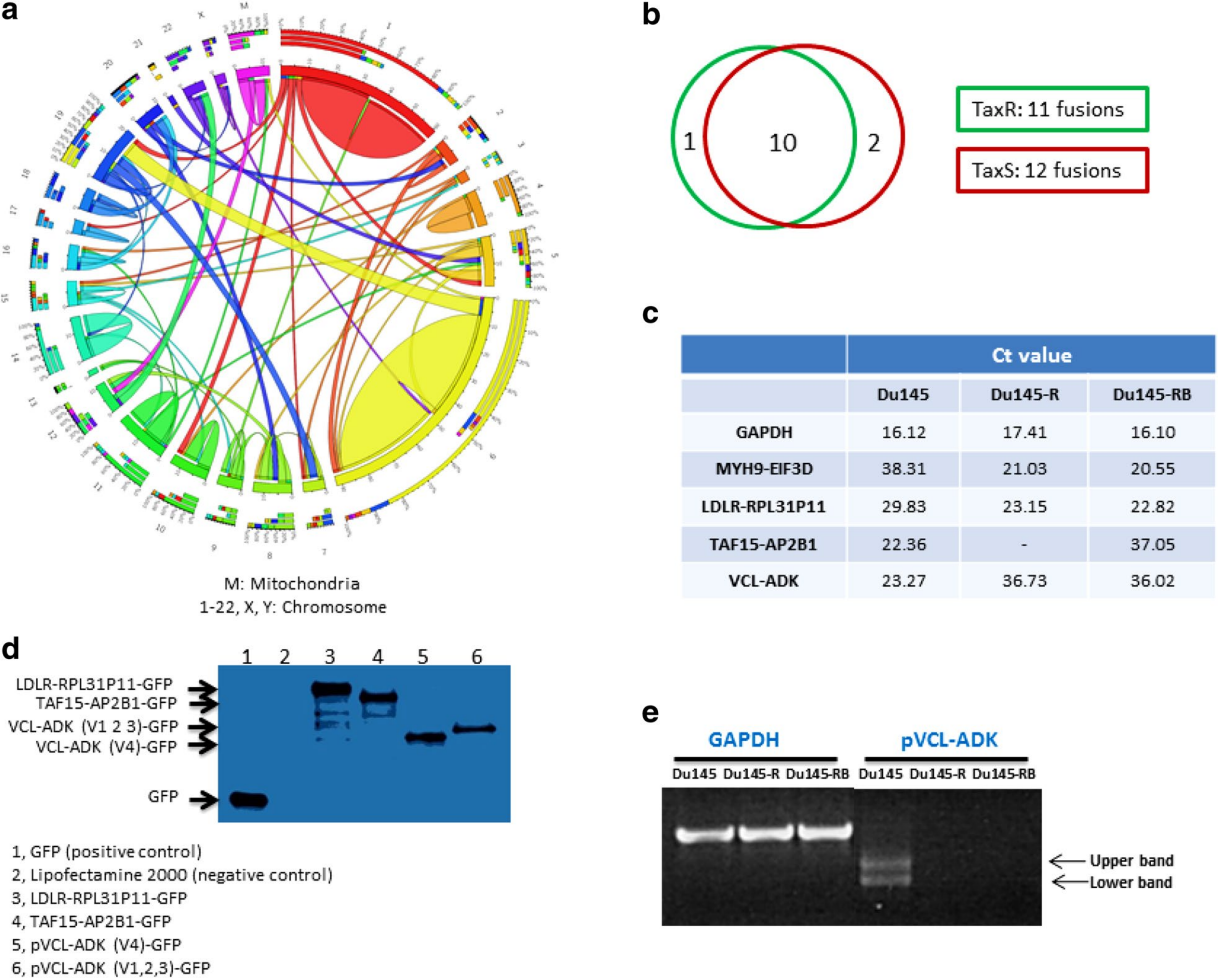
Landscape and validation of fusion candidates. **a** Circos plot of the genomic landscape of gene fusions discovered by RNAseq. **b** *Venn diagram* analysis of 13 fusions which could be validated by PCR. **c** qPCR validation of fusions in Du145, Du145-R and Du145-RB. **d** Fusion proteins detected in transfected HEK293 cells by Western blot. **e** Two different fusion transcript types, that is between VCL-ADK variants 1, 2, 3 and VCL-ADK variant 4

Figure 3a showed that all chromosomes were involved in gene fusion except chr 21 and chr Y. The two largest fusion groups were distributed in chr1 (27, 14.3 %) and chr6 (44, 23.3 %), and most of the fusions were intra-chromosomal (26 out of 27 in chr1; 39 out of 44 in chr6). Of the 16 chosen fusion candidates (3 of them could not be validated by PCR), 10 of them were commonly expressed in all 3 cell lines, one expressed only in TaxR cell line (MYH9-EIF3D) and 2 specifically in TaxS cell line (TAF15-AP2B1, VCL-ADK) (Table 2; Fig. 3b). Among 10 commonly expressed fusions, two were up-regulated in TaxR cell lines compared to TaxS cell line (LDLR-RPL31P11, SRGAP2P2-SRGAP2). Eight out of 16 were predicted to be in-frame suggesting their potential to produce functional fusion proteins (Table 2).

Four fusion candidates were validated by qPCR in Du145, Du145-R and Du145-RB cells, as well as at the protein level by western blot in plasmid transfected HEK293 cells (Fig. 3c, d), but their translation into protein could not be validated by western blot in Du145, Du145-R and Du145-RB cell lines.

Interestingly, when we validated the VCL-ADK fusion candidate by PCR, we found that there were two bands in the same PCR lane (Fig. 3e). Sanger sequencing results showed that both of the two bands were VCL-ADK fusions. The upper band was a fusion between VCL and ADK variant 1, 2 and 3, while the lower band was another fusion with ADK variant 4. Western blot showed that both fusions (VCL-ADK variant 1, 2, 3 and VCL-ADK variant 4) could be detected as protein in plasmid transfected HEK293 cell lines (Fig. 3d).

### Identification of stably up- or down-regulated genes in the TaxR cell lines

Using gene expression of parental Du145 (TaxS) cells as a baseline, we identified 453 up-regulated and 473 down-regulated genes in the Du145-R cells, and 483 up- and 365 down-regulated genes in the Du145-RB cells (Additional file 4). In addition, we found 216 genes with significantly different expression levels between DU145-RB and DU145-R. These 216 genes were presumably not related to the development of docetaxel resistance. By matching the three gene lists we further identified 615 (329 up-regulated and 286 down-regulated) genes that were shared by both DU145-R and DU145-RB as compared with DU145 (Additional file 4). These genes were thought to have stable expression changes after acquiring resistance to docetaxel. Of the 615 genes, the 40 most up- and down-regulated in the TaxR cell lines were chosen for verification by RT-PCR and 37/40 (92.5 %) were confirmed (Additional file 5).

Information about the most-differentially-expressed genes is shown in Additional file 6. The second most up-regulated gene was ABCB1, which encodes an ATP-dependent drug efflux pump that mediates the development of resistance to anticancer drugs (Gottesman et al. 2002). The average fold changes (log) in TaxR cell lines were up to 8.9 and 10.2 in up-regulated genes and down-regulated genes, respectively. The largest functional group was transcription factors (Additional file 7). Twenty-one oncogenes and 16 translocated cancer genes were also among the enriched functional groups in the set of 615 stably differentially-expressed genes.

**Fig. 4:**
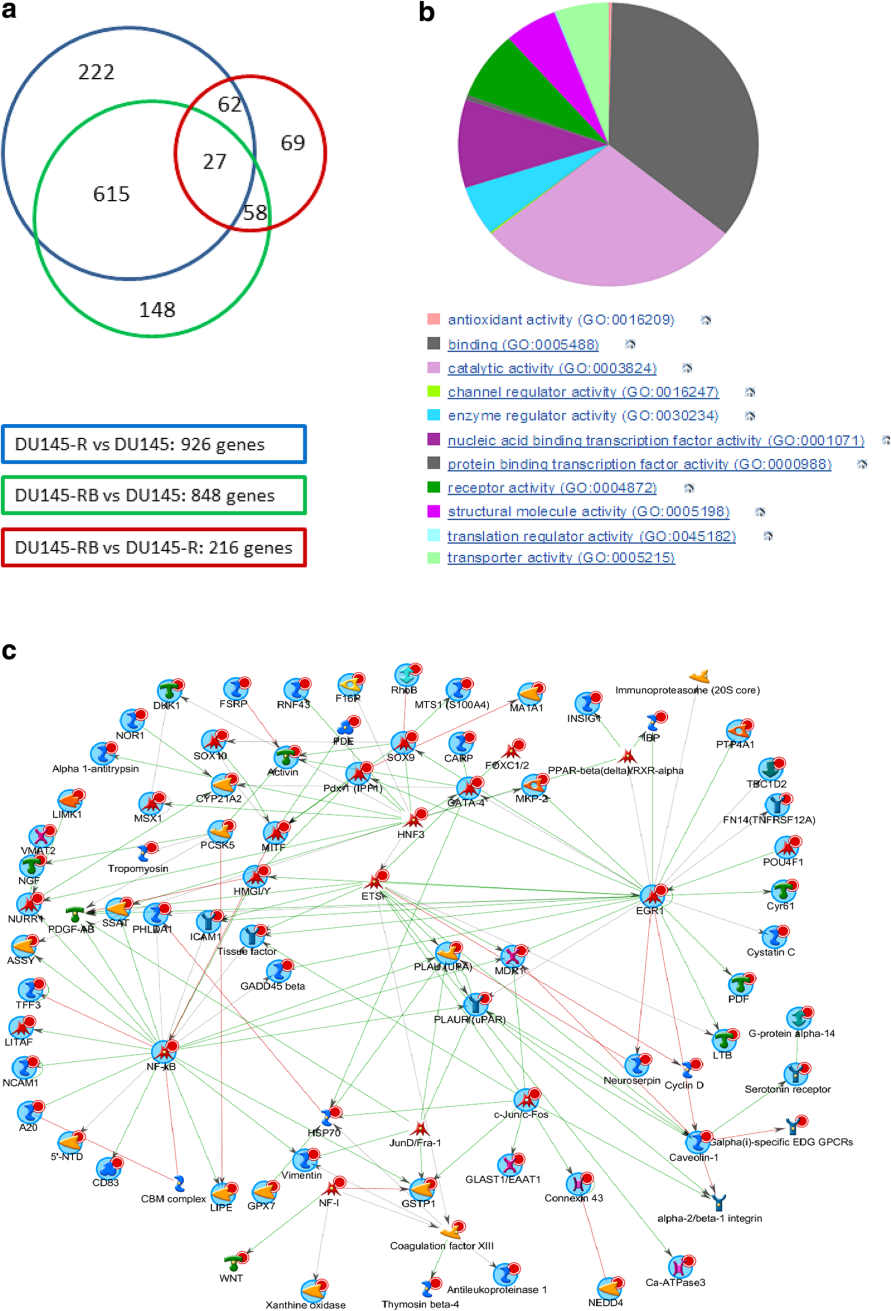
NGS analysis in TaxS and TaxR prostate cancer cell lines identifies gene expression changes and pathway networks involved in docetaxel resistance. **a** *Venn diagram* of overlap of significantly differently regulated genes by matching 3 gene lists. **b** *Pie chart* of 615 genes with stable gene expression changes in TaxR cells lines by Panther (www.pantherdb.org). **c** GeneGo (Thomsom Reuters) network analysis of the most deregulated genes in TaxR cell lines

The 615 most significantly deregulated genes were put into the Panther Online tool (www.pantherdb.org), which yielded 528 functional hits distributed on 11 GO-terms, where the two largest groups were Binding (GO: 0005488) and Catalytic Activity (GO: 0003824) (Fig. 4b). Thomson Reuters was employed to analyze enriched networks of expression changing genes and showed that the NF-κb, EGR1 (Early Growth Response 1) and ETS (ETS family of transcription factors) were the three most enriched networks in the docetaxel resistant cells (Fig. 4c and Additional file 8). PLAU and PLAUR (Plasminogen Activator, Urokinase Receptor), a ligand—membrane receptor pair, are the only ‘Convergence hubs’ and MDR1 (ABCB1) was connected to all three pathways.

Next generation sequencing data of PC3 and LNCaP, two docetaxel-sensitive cell lines similar to Du145, were added into further analysis. Multivariate modeling with SIMCA resulted in a model, which separated all TaxS and TaxR cell lines into two classes and extracted those genes that contributed most to the model (Table 3).

**Table 3:** Genes contributed most to separate prostate cancer cell lines into TaxR and TaxS classes

Gene symbol	Full name	M1.VIPpred
ABCB1	ATP-binding cassette, sub-family B (MDR/TAP), member 1	1.48587
GPSM2	G-protein signaling modulator 2	1.48037
IL31RA	Interleukin 31 receptor A	1.47976
LIMK1	LIM domain kinase 1	1.47921
GRK5	G protein-coupled receptor kinase 5	1.47789
SKAP1	Src kinase associated phosphoprotein 1	1.47782
ST6GALNAC5	Sialyltransferases	1.4765
C9orf125	Transmembrane protein 246	1.47636
ICAM1	Intercellular adhesion molecule 1	1.47543

M1.VIPpred, score shows the contribution to the SIMCA model. All 9 genes in this table were up-regulated in the TaxR cells

When comparing the list of 615 stably up- or down-regulated gene lists with the fusion gene list, we found 6 genes in common (Table 4), all of which were up-regulated.

**Table 4:** Fusion genes whose expression levels were also up-regulated in TaxR cell lines

Gene	Full name	Fold up-regulation	Fusion partner	Full names of fusion partners
HIVEP2	Human immunodeficiency virus type I enhancer binding protein 2	1.4	JA040725	JA040725
HMGA1	High mobility group AT-hook 1	1.8	BTNL8	Butyrophilin like 8
PTRF	Polymerase I and transcript release factor	1.7	ABCA9	ATP binding cassette subfamily A member 9
RPL31P11	Ribosomal protein L31 pseudogene 11	3.9	LDLR	Low density lipoprotein receptor
VCL	Vinculin	2.3	ADK	Adenosine kinase
VIM	Vimentin	4.9	SYCP1	Synaptonemal complex protein 1

Fold up-regulation, log_2_ fold expression change of genes in column 1 comparing TaxS cell line and TaxR cell lines

## Methods

### Prostate cancer cell lines

Table 5 summarizes the prostate cancer cell lines used in this study. LNCaP, PC3 and DU145 cell lines were originally ordered from the ATCC (American Type Culture Collection). Du145 was cultured in medium containing docetaxel (from low concentration to high concentration, increased gradually) for one year, until Du145 acquired docetaxel resistance (Du145-R). We also cultured Du145-R in normal medium without docetaxel for one month (Du145-RB) to see if it would revert to docetaxel sensitive again (Kharaziha et al. 2015). DU145-RB was frozen after one month cultured without docetaxel. Every time when we needed to use DU145-RB, we would thaw and culture it in normal medium (without docetaxel) and the culture time would not extend 4 weeks. While, DU145-R cells were always cultured in medium with 1000 ng/ml docetaxel. DU145, PC3 and LNCaP cells were cultured in medium without docetaxel.

**Table 5:** Prostate cancer cell lines which were analyzed by whole transcriptome sequencing

Cell line	Androgen-dependent	Docetaxel-sensitive	Triplicates
LNCaP	Yes	Yes	Yes
PC3	No	Yes	Yes
DU145	No	Yes	Yes
DU145-R	No	No	Yes
Du145-RB	No	No	Yes

Androgen-dependent, cell line is sensitive to hormone treatment (Yes) or not (No). Docetaxel-sensitive, cell line is sensitive to docetaxel (Yes) or not (No). Triplicates, all cell lines were triplicates when sent to be sequenced

### RNAseq

Total RNA from prostate cancer cell lines was isolated by TRIzol (Invitrogen, Catalog #15596018) and extracted by subsequent phenol/chloroform. RNase-free DNase set (Qiagen, Catalog #79254) was used to remove DNA by DNase digestion. RNA quality was controlled by RNA Integrity Number (RIN) analysis by Agilent 2100 Bioanalyzer System. Total RNA samples were sent to SciLifeLab, Stockholm, Sweden and polyA selection was done at SciLifeLab. Samples were clustered on cBot and sequenced on HiSeq 2000 according to manufacturer’s instructions. Between 16.0 and 76.3 million reads were obtained per sample sequenced on HiSeq 2000.

### Variant calling method

Removal of PCR duplicates was performed with Picard (picard.sf.net). After that the reads were extracted from bam file, imported into CLC Genomics workbench (CLC, Aarhus, Denmark) and aligned to the human reference genome (build 37p5) using Large Gap Read Mapping. Variant calling was conducted using Probabilistic Variant Detection tool within CLC Genomics workbench. The following criteria were applied for variant calling: (1) ignore non-specific matches, (2) Minimum coverage, and (3) Variant probability 90. The variations were filtered out if detected in any of 190 control exomes from non-cancer patients, or were dbSNP v137 reported SNPs, with a population frequency higher than 1 % in dbSNP v137. The variants were annotated according to their overlap with genes and transcripts (UCSC, refSeq at http://genome.ucsc.edu/, and Sanger cancer census gene at http://cancer.sanger.ac.uk/cancergenome/projects/census/), conservation scores (UCSC), segmental duplications (UCSC), exon number, splice sites, amino acid change, cosmic database v63, ClinVar (a database of mutations and their clinical relevance at ftp://ncbi.nlm.nih.gov/pub/clinvar/), dbSNP v137 and predictions from Provean (http://provean.jcvi.org), Sift (http://sift.jcvi.org) and Polyphen (http://genetics.bwh.harvard.edu/pph2/bgi.shtml).

### Analysis of differentially expressed genes

We analyzed RNAseq data according to a published TopHat and Cufflinks protocol (Trapnell et al. 2012). In summary, we used TopHat to align reads to the reference genome, Cufflinks to assemble and obtain expression values for all transcripts, Cuffdiff for testing differential expression of genes and transcripts and finally the CummeRbund R package for downstream analysis and visualization.

### Fusion detection method

We used ChimeraScan, which aligns paired-end reads to a reference genome-transcriptome with Bowtie in an iterative process where read pairs that could not be aligned were trimmed into smaller fragments and realigned (Iyer et al. 2011). ChimeraScan uses a filter to avoid false-positive chimeras.

### Statistical analysis

The online services Panther (http://www.pantherdb.org) and Thomson Reuters were applied for functional enrichment analysis (Mi et al. 2013; Huber-Keener et al. 2012). Fusion transcripts from Du145, Du145-R and Du145-RB were visualized by Circos online (http://mkweb.bcgsc.ca/tableviewer) (Krzywinski et al. 2009). OPLS-DA model was established by SIMCA software, and 2 classes (TaxS and TaxR) were set in the model to obtain VIP scores by whichvariables (genes) are sorted based on importance (contribution to the model) of genes (Bylesjo et al. 2006).

### PCR and qPCR validation

Total RNA was isolated from cell lines by TRIzol (Invitrogen, Catalog #15596018) according to the manufacturer’s instructions. Cloned AMV First-Strand Synthesis Kit (Life Technologies, Catalog #12328) was used to transcribe mRNA to cDNA.

PCR primers for fusion validation were designed according to the sequence of fusion transcripts. Forward primer was located on the 5′ gene of the fusion gene and reverse primer on the 3′ gene of the fusion gene. Primers for validation of mutations covered mutation points. PCR was conducted using Platinum Taq DNA polymerase (Life Technologies, Catalog #10966018) and was followed by Sanger sequencing (conducted by Eurofins Genomics). To differentiate gene expression levels of selected genes, 20–32 amplifying cycles were used based on gene expression level.

qPCR primers for fusion validation were purchased from Applied Biosystems (Custom plus TaqMan RNA Assays). LightCycler 480 Probes Master was used combined with TaqMan primer on LightCycler 480 instrument from Roche according to manufacturer’s instructions.

### Plasmid construction and western blot validation

PCR product was ligated into multiple cloning sites of pCMV-AC-GFP after digestion of restriction enzymes, Sgf I and Mlu I. pCMV-AC-GFP was purchased from ORIGENE (Catalog #PS100010), Sgf I enzyme from NEB (Catalog #R0630S), Mlu I enzyme from NEB (Catalog #R0198S), and T4 ligase from Promega (Catalog #M180A). We transfected HEK293 cells with constructed plasmid 48 h before collecting cells in lysis buffer. Western blot experiments were conducted using these cell lysates. Anti-TurboGFP antibody was purchased from Evrogen (Catalog #AB513).

## Discussion

We have identified 42 genes with specific and stable mutations in TaxR cells. The functions of these genes may support their importance in the development of docetaxel resistance. Among these genes, SMAD4 is a co-activator and mediator of signal transduction by TGF-beta and acts as a tumor suppressor. Experiments have shown that SMAD4 inactivation promotes drug resistance in cancer (Zhang et al. 2014; Raz et al. 2014). ABCA2 is a member of ATP-binding cassette (ABC) transporters that transports many kinds of small molecules through membranes and is involved in drug resistance in leukemia cell lines (Dharmapuri et al. 2015).

Approximately 50 % of prostate cancer has primary resistance to docetaxel treatment. The other half is sensitive to docetaxel but eventually develops secondary (acquired) resistance (Marin-Aguilera et al. 2012). In this study, 34 out of the 42 mutations discovered in the resistant cell lines can be found in tumor samples from patients (Table 1), implicating that primary and acquired resistance may share the same molecular mechanism(s). In the case of primary resistance, most cancer cells carry the resistant genomic changes before the treatment, whereas for acquired resistance, just a few cancer cells carry these resistant genomic changes before treatment. By treatment selection or new mutational events, most cancer cells become carriers of resistant genomic changes. This hypothesis can be further tested in studies using tumor samples from patient cohorts with data of docetaxel treatment.

The four fusion transcripts (listed in Fig. 3c: MYH9-EIF3D, LDLR-RPL31P11, TAF15-AP2B1, VCL-ADK) could be detected by PCR and qPCR in the cell lines, but their translation into protein could not be validated by western blot in Du145, Du145-R and Du145-RB, probably due to the low expression of the fusion proteins. Fusion transcripts could be translated into protein in stably transfected HEK293 cells analyzed by western blot. Moreover, several genes involved in the fusion events have shown important functions in cancer development. TAF15, a member of the FET family, has been found rearranged with various transcription factors with cancer promoting functions in sarcomas as well as in rare hematopoietic and epithelial cancers (Kovar 2011). MYH9 is a member of the myosin superfamily and its function is related to migration, invasion and metastasis of cancer cells. EIF3D is associated with cell cycle regulation and motility of prostate cancer cells (Gao et al. 2015). MYH9 fusion proteins have been found in anaplastic large cell lymphoma and one example is the MYH9-ALK fusion protein that has tyrosine kinase activity in vivo (Lamant et al. 2003). The MYH9-USP6 detected by a previous study and MYH9-EIF3D found in the present study have the same fusion point in MYH9. MYH9, which is located in the 5′ part of the fusion product, functions as a regulator to manipulate gene expression and function of USP6, as well as EIF3D (Erickson-Johnson et al. 2011). These functional implications may encourage further verification by using tumor samples from the patients.

When we compared the expression of DU145-RB and DU145-R, we found 216 genes that were differently expressed. We tested that DU145-RB was still docetaxel resistant, indicating these genes were not involved in maintaining docetaxel resistance of the two resistant cell lines. As expected, ABCB1 (MDR1) was confirmed as one of the top 10 differentially expressed genes that could separate TaxR from TaxS cells. Its functional importance was further supported by its connection with the NF-κb, EGR1 and ETS pathways (Fig. 4). ABCB1, which shows overexpression in some cancers, is involved in a common resistance mechanism. However, limited studies showed significant connection between ABCB1 and clinical outcomes, such as survival (Shaffer et al. 2012), indicating the importance of other molecular and biological changes. Researchers and pharmaceutical companies are trying to circumvent this strategy and find new potential genes or pathways to overcome resistance in cancer.

TGPSM2 and GRK5 are members of G-protein signaling pathway important in cancer progression. SKAP1 encodes a src kinase associated phosphoprotein 1 and is a member of the Ras signaling pathway and B cell receptor signaling pathway. LIMK1 is a serine/threonine kinase associated with the cytoskeletal structure in many cellular processes, and may have importance in the sensitivity of lung cancer and osteosarcoma cells to chemotherapy treatment (Chen et al. 2013; Zhang et al. 2011). The analysis further showed that PLAU and PLAUR (Plasminogen Activator, Urokinase Receptor), a pair of ligand and membrane receptor, constituted the only ‘Convergence hub’ by statistical analysis using the Thomson Reuters software. This novel finding may suggest that they may play a unique role in docetaxel resistance. It would be interesting to further study if they alone or, together with other important genomic findings in this study, can be further verified as important biomarkers to predict primary docetaxel resistance. Most importantly, they can even become attractive targets for the development of new drugs to overcome both primary and acquired docetaxel resistance.

## Conclusion

The present study found both previous and novel mutations, genes with altered expression levels, and fusion proteins in docetaxel resistant prostate cancer cell lines, and provide some understanding of acquired docetaxel resistance at the gene transcription level. If some of these changes can be further verified with importance in primary resistance, they can be considered as predictive biomarkers for docetaxel treatment as well as targets for the development of new treatments to overcome the docetaxel resistance.

## Additional files

**Additional file 1.** Mutation in all TaxS and TaxR cell lines.

**Additional file 2.** Fusions in all TaxS and TaxR cell lines.

**Additional file 3.** PCR validation of fusion candidates.

**Additional file 4.** Expression changing genes in TaxR cell lines.

**Additional file 5.** PCR validation of expression changing genes.

**Additional file 6.** Most differentially-expressed genes in TaxR cell lines.

**Additional file 7.** Distribution of 615 genes with stable expression level changes in TaxR cell lines.

**Additional file 8.** Hubs in the network analysis.
