# Erratum to: Identification of mutations, gene expression changes and fusion transcripts by whole transcriptome RNAseq in docetaxel resistant prostate cancer cells

**DOI:** 10.1186/s40064-016-3759-z

**Published:** 2016-12-08

**Authors:** Yuanjun Ma, Yali Miao, Zhuochun Peng, Johanna Sandgren, Teresita Díaz De Ståhl, Mikael Huss, Lena Lennartsson, Yanling Liu, Monica Nistér, Sten Nilsson, Chunde Li

**Affiliations:** 1 Department of Oncology-Pathology, Karolinska Institutet, Stockholm, Sweden; 2 Department of Obstetrics and Gynecology, Beijing University People’s Hospital, Beijing, China; 3 SciLifeLab (Science for Life Laboratory), Stockholm, Sweden; 4 Clinical Pathology/Cytology, Karolinska University Hospital, Stockholm, Sweden; 5 Department of Clinical Oncology, Karolinska University Hospital, Stockholm, Sweden

## Erratum to: SpringerPlus (2016) 5:1861 DOI 10.1186/s40064-016-3543-0

After the publication of our article (Ma et al. 2016), we became aware of an error in the “Fusion transcript detection and validation” section of our Results.

“Two gene fusions had been found by previous studies: UBE2L3-KRAS (Wang et al. 2011) expressed in all three cell lines and TAF15-AP2B1 (http://54.84.12.177/PanCanFusV2/Fusions!fusion) specific expressed in Du145 (Additional file 3). The other fourteen fusions were novel discovered.” 

Should read:

“Two gene fusions had been found in prostate cancer in previous studies (Wang et al. 2011; Klijn et al. 2015): UBE2L3-KRAS and C14orf166-SLC25A21 were expressed in all three cell lines in our study (Additional file 3). The other fourteen fusions are reported here for the first time in prostate cancer.”
