# Supplementary figures and images for: Identification of mutations, gene expression changes and fusion transcripts by whole transcriptome RNAseq in docetaxel resistant prostate cancer cells
Source: Springerplus. 2016 Oct 24;5(1):1861. doi: 10.1186/s40064-016-3543-0 (PMC5078122; doi:10.1186/s40064-016-3543-0)

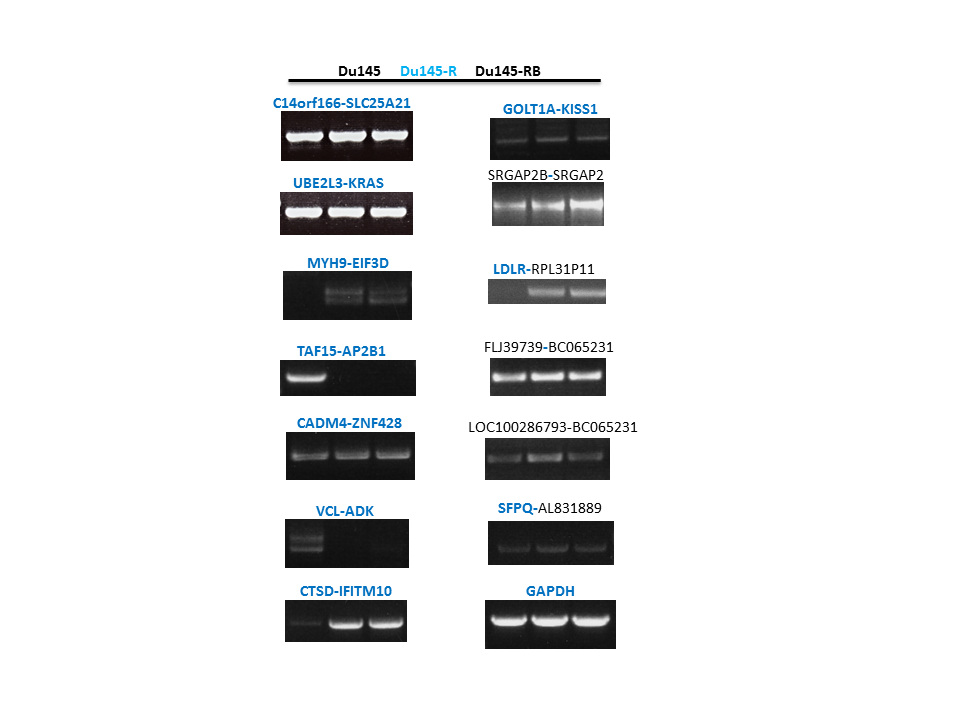

Supplement: Supplementary file 3 — Additional file 3. PCR validation of fusion candidates. [file 40064_2016_3543_MOESM3_ESM.tif]
