# Supplementary material for: Identification of mutations, gene expression changes and fusion transcripts by whole transcriptome RNAseq in docetaxel resistant prostate cancer cells
Source: Springerplus. 2016 Oct 24;5(1):1861. doi: 10.1186/s40064-016-3543-0 (PMC5078122; doi:10.1186/s40064-016-3543-0)
Supplement: Supplementary file 5 — Additional file 5. PCR validation of expression changing genes. [file 40064_2016_3543_MOESM5_ESM.pptx]

## Slide 1
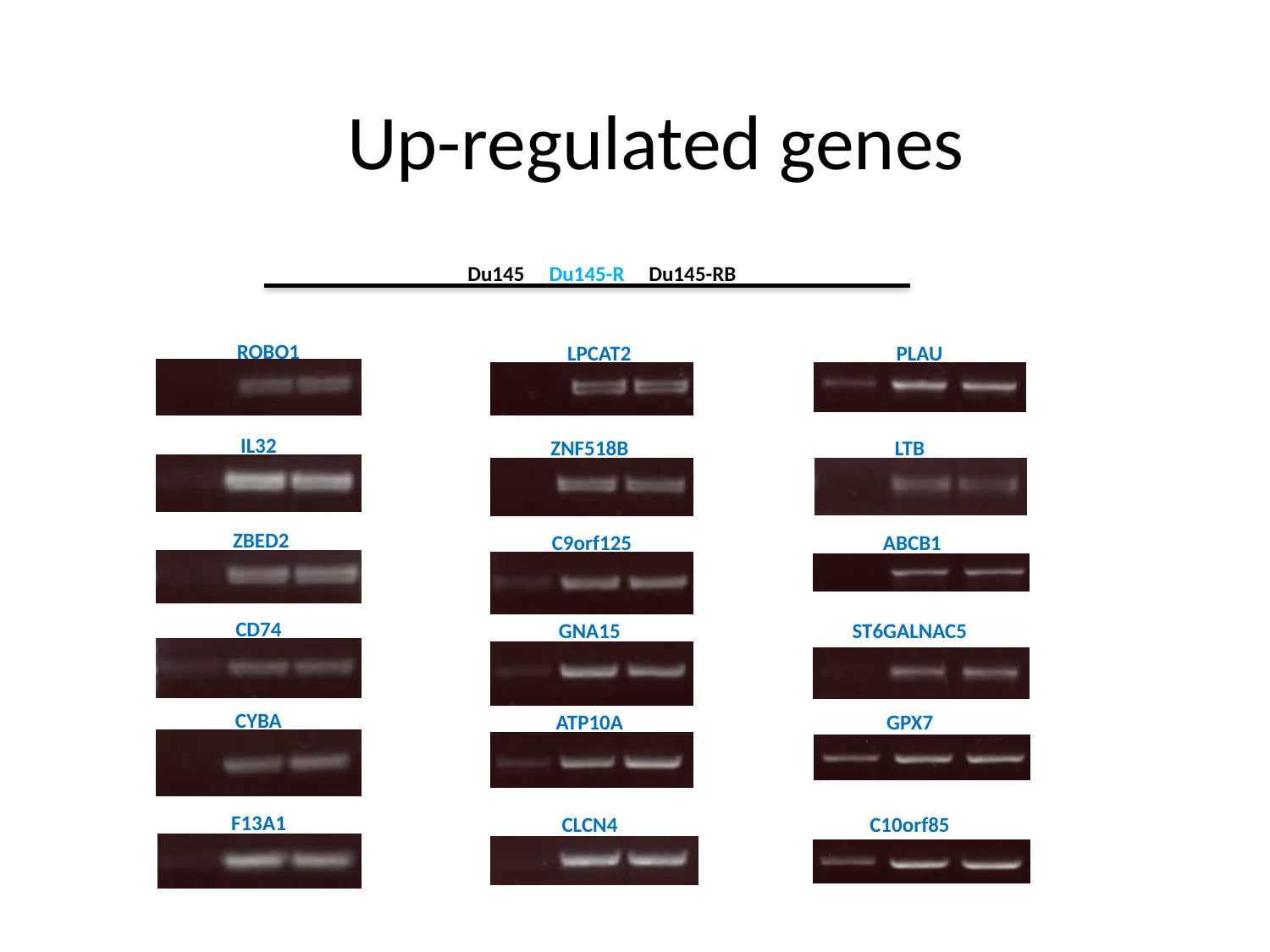

#
Up-regulated genes
Du145 Du145-R Du145-RB
 ROBO1
 LPCAT2
 PLAU
IL32
ZNF518B
LTB
 ZBED2
 C9orf125
 ABCB1
CD74
GNA15
ST6GALNAC5
CYBA
ATP10A
GPX7
F13A1
CLCN4
C10orf85

## Slide 2
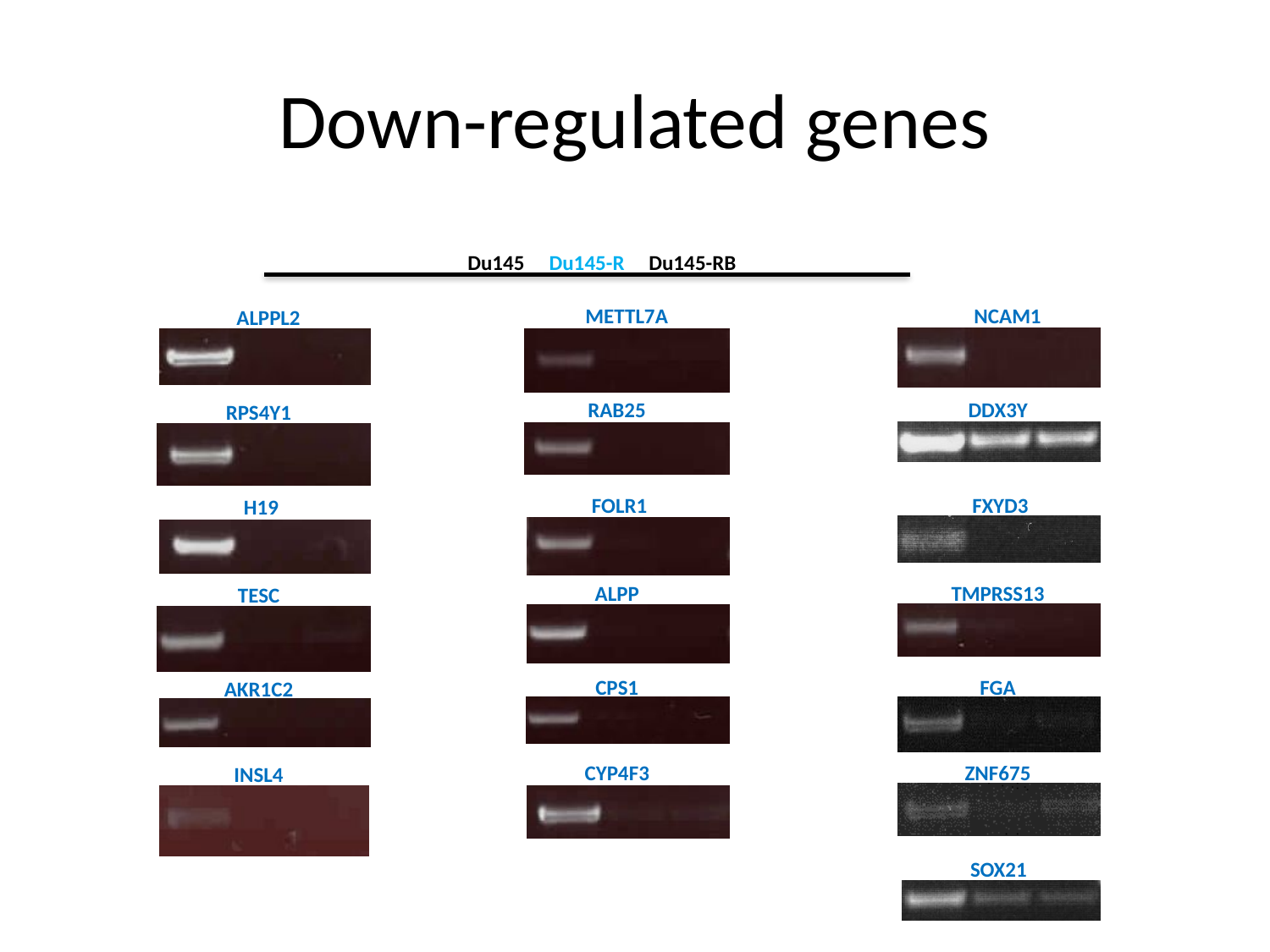

# Down-regulated genes
Du145 Du145-R Du145-RB
 METTL7A
 NCAM1
 ALPPL2
RAB25
DDX3Y
RPS4Y1
 FOLR1
 FXYD3
 H19
ALPP
TMPRSS13
TESC
CPS1
FGA
AKR1C2
CYP4F3
ZNF675
INSL4
SOX21
